# Naloxone Knowledge, Carrying, Purchase, and Use

**DOI:** 10.1001/jamanetworkopen.2024.62698

**Published:** 2025-03-03

**Authors:** Mireille Jacobson, David Powell

**Affiliations:** 1 University of Southern California, Los Angeles; 2 RAND, Arlington, Virginia

## Abstract

**Importance:**

Widespread naloxone access is a key policy response to the opioid crisis. Naloxone availability is typically estimated from pharmacy sales, which exclude naloxone provided by community organizations, hospitals, and clinics, or sold over-the-counter.

**Objective:**

To estimate naloxone knowledge, carrying, purchase, and use among US adults.

**Design, Setting, and Participants:**

This survey study included noninstitutionalized adults aged 18 years and older from a national sample and a sample self-reporting opioid dependence. Respondents answered online questions between June 7 and June 29, 2024, about naloxone knowledge, carrying, purchase, and use.

**Exposures:**

Opioid misuse, risk of overdose, risk of overdose by person known to respondent.

**Main Outcomes and Measures:**

Naloxone knowledge, prevalence of naloxone carrying, purchase, and administration.

**Results:**

The survey included 1515 individuals from a national sample (median [IQR] age, 45 [33-58] years; 770 women [50.8%]; 215 Black [14.2%], 1087 White [71.8%]) and 512 who self-reported opioid dependence. In the national sample, 50 respondents (3.3%) reported opioid dependence, yielding 562 respondents reporting opioid dependence (median [IQR] age, 41 [35-48] years; 404 female [70.2%]; 17 Black [3.0%], 494 White [87.9%]). Overall, 1164 respondents—700 (46.2%) in the national sample and 500 (89.0%) reporting opioid dependence—had heard of naloxone and correctly identified its purpose. One hundred sixty participants (10.6%) in the national sample and 340 participants (60.5%) in the sample reporting opioid dependence reported carrying naloxone. Among those reporting they were “very likely to overdose,” 22 respondents (31.0%) in the national sample and 31 (73.8%) with opioid dependence reported carrying naloxone. Among those who know someone very likely to overdose, 43 participants (25.4%) in the national sample and 190 participants (70.1%) reporting opioid dependence reported carrying naloxone. Among those who ever carried naloxone, 108 (42.4%) in the national sample and 97 (22.6%) reporting opioid dependence had ever purchased naloxone. Overall, 128 respondents (8.4%) in the national sample and 267 respondents (47.5%) reporting opioid dependence reported administering naloxone to someone else while 93 (6.1%) in the national sample and 221 (39.3%) reporting opioid dependence reported being administered naloxone.

**Conclusions and Relevance:**

In this survey study of naloxone, most respondents reporting opioid dependence correctly identified naloxone’s purpose and carried it. Most naloxone carried was not purchased, suggesting a need for new ways, including rapid online surveys, to monitor naloxone possession.

## Key Points

**Question:**

What percentage of US adults, including those at heightened risk of opioid overdose, have carried, purchased, or used naloxone?

**Findings:**

In this survey study of a national sample of 1515 adults with 512 adults oversampled because of self-reported opioid dependence, 10.6% of the national sample and 60.5% of respondents reporting opioid dependence currently carry naloxone. Among those who have ever carried naloxone, 42.4% in the national sample and 22.6% reporting opioid dependence had ever purchased naloxone.

**Meaning:**

In this study, naloxone carrying rates were high, particularly among adults self-reporting opioid dependence; however, most naloxone was not purchased, highlighting the importance of survey-based approaches to monitor possession.

## Teaser

This survey study of US adults examines awareness of and carrying rates for naloxone for treatment of opioid overdose.

## Introduction

Over 75% of the more than 100 000 drug overdoses in the US in 2023 involved opioids.^1^ While effective medications to reverse opioid overdoses exist, adoption at scale can be difficult to achieve.^2,3^ Over the past decade, policymakers have worked to increase access to naloxone, an opioid antagonist that is highly effective at reversing opioid overdose.^4,5,6^ Every state now permits pharmacies to dispense naloxone without a patient-specific prescription.^4,7,8^ In 2023, 2 naloxone products were made available over-the-counter (OTC), further facilitating consumer access.^9,10^ However, important data limitations make it difficult to track the proportion of people who possess or carry naloxone.

Naloxone access is generally quantified using pharmacy dispensing data and, to a lesser extent, insurance claims.^11,12,13,14,15,16^ Both data sources effectively restrict to purchased naloxone and do not capture naloxone provided through community-based organizations, first responders, hospitals, clinics, or other nonpharmacy settings. They also do not capture OTC naloxone sales, which a 2024 secret shopper study in North Carolina found increased retail pharmacy availability.^17^ Naloxone distribution data can provide a more accurate view of the volume of naloxone supplied across pharmaceutical and nonpharmaceutical settings,^18^ but these data are not readily available. Some studies and reports have provided information on naloxone distribution for specific areas or states.^19,20^ Importantly, even such rich data cannot shed light on individual carrying or use of naloxone overall or based on risk of overdose.

The objective of this study was to provide national estimates of naloxone knowledge, carrying, purchase, and administration. We developed and fielded an online survey to a national sample of adults and an oversample of adults who self-reported currently or ever having “opioid dependence,” a term used by the survey firm in a health screener. We oversampled those reporting opioid dependence to characterize knowledge and carrying among people at higher risk of overdose and thus higher potential benefit from naloxone than the general population.

## Methods

Between June 7 and June 29, 2024, we surveyed adults about their knowledge of and experience with both opioids and naloxone. The study was approved by the RAND Corporation’s Human Subjects Protection Committee and the University of Southern California’s institutional review board. We informed participants of the sensitive nature of the survey and required online consent before survey completion (eAppendix 1 in Supplement 1). This study followed the American Association for Public Opinion Research (AAPOR) reporting guidelines.

### Participants, Setting, and Survey Design

We employed an internet panel from Respondi, a well-established survey research firm, to recruit 2 cross-sectional samples of adults aged 18 years and older—a national sample and a sample of persons self-reporting current or prior opioid dependence. Respondi panels, which are maintained through active recruitment and validated authentication, have been used in many published studies.^21,22,23,24,25,26,27,28,29^ Panel members receive small incentives for survey participation. For the national sample, we set recruitment quotas by sex (male, female), race (White, race other than White), age-group (aged 18 to 29 years, 30 to 39 years, 40 to 49 years, 50 to 59 years, 60 years and over), and region (Northeast, Midwest, South, West) to achieve national representation on these important demographics. The national survey remained in the field until 1515 surveys were completed, a number determined by the cost of a completed survey and available funding.

We separately recruited respondents who had previously indicated opioid dependence (eAppendix 2 in Supplement 1). We screened out of this oversample respondents who no longer chose opioid dependence among a set of 9 possible current or prior health conditions. The order of conditions, which included alcohol dependence, asthma, cancer, cardiovascular disease, chronic kidney disease, chronic liver disease, depression, and diabetes, was randomized. Respondents could select multiple conditions or none of the above. The opioid dependence sample remained in the field until 512 surveys were completed, also determined by cost and funding. For analysis, we included the national sample participants who indicated opioid dependence in the oversample.

We excluded respondents who failed an attention check (746 nationally; 147 with opioid dependence), a commonly used approach to screen out individuals unthinkingly completing surveys,^30,31^ or who did not complete the survey (29 nationally; 17 with opioid dependence) (eAppendices 3 and 4 in Supplement 1). eFigure 1 in Supplement 1 provides flow diagrams showing sample construction.

### Study Measures

Our primary study measures captured the percentage of respondents who: (1) had knowledge of naloxone, (2) currently carry naloxone, (3) ever purchased naloxone; (4) were ever administered naloxone, and (5) had ever administered naloxone to someone else. Naloxone knowledge was based on 2 questions. First, respondents were asked if they had heard of the “overdose reversal medication” naloxone or the brand name version, Narcan. Those responding negatively to this question were automatically coded as not having naloxone knowledge and were informed that naloxone can be used to reverse overdoses involving opioids. Those who responded affirmatively were tested about the kind of overdose naloxone can reverse. Possible answers, presented in random order, included “any overdose”; “opioid overdose (heroin, oxycodone, methadone)”; “amphetamine overdose (Adderall)”; “cocaine overdose”; or “I don’t know.” Only respondents who chose opioid overdose were coded as having knowledge of naloxone. All respondents were shown the correct answer after question completion.

Respondents were asked whether they had “previously carried” naloxone, “currently carry it occasionally,” “currently carry it most or all of the time,” or “have never carried” naloxone. Respondents selecting either of the 2 currently carrying options were coded as currently carrying naloxone; those selecting previously or currently carried options were coded as ever carrying naloxone. Respondents were asked whether and when they had “ever administered naloxone to another person” and, separately, whether and when anyone had ever administered naloxone to them. They were also asked where they obtained the naloxone they currently or previously carried and if they had ever purchased naloxone.

To understand potential need for naloxone, we asked about opioid use and self-assessed risk of opioid overdose. Specifically, we asked about use in the past 12 months of prescription opioids, any nonprescription opioids, and illicitly made fentanyl. We also asked whether any prescription opioids were used in the past 12 months “in a way that a doctor did not direct you to use.” Respondents were asked to rate as unlikely, somewhat likely, likely, or very likely the possibility that “someone you know might overdose from opioid use” and, separately, that “you might overdose from opioid use.”

Respondents provided basic demographic information from multiple choice survey questions. The demographic questions captured gender, age, education, race (American Indian or Alaska Native, Asian American or Pacific Islander, Black, White, or other [given a free-text field, most respondents reported “Hispanic” or “Latino”]), ethnicity, and state of residence. Race and ethnicity, like all the demographic data, were self-reported.

### Statistical Analyses

We computed the proportion of respondents who knew naloxone’s purpose and those who have carried, purchased, were administered, and administered naloxone to someone else in both the national and the opioid dependence samples. In addition to proportions overall, we computed proportions based on prior opioid use and self-assessed risk of opioid overdose for the respondent and for someone they know. We also computed 95% CIs for binomial data.^32^ We analyzed the sensitivity of findings to sample exclusion restrictions based on the attention check and incomplete survey responses. All analyses were performed using Stata MP, version 18 (StataCorp LLC).

## Results

### Sample Characteristics

The national sample included 1515 respondents (median [IQR] age, 45 [33-58] years; 770 identified as women [50.8%]; 215 reported Black [14.2%], 1087 White [71.8%] race; 256 [16.9%] reported Hispanic ethnicity; and 560 [37.0%] reported living in a state in the South Census region of the US) (Table). Based on the May 2024 Current Population Survey (CPS), the national sample looked similar based on gender, race, Hispanic ethnicity, and region of the country compared with the US population aged 18 years and over (eTable 1 in Supplement 1). The sample differed most clearly based on education: the percentage of respondents with no college experience was low relative to the population, a difference that may overstate naloxone knowledge and subsequent downstream outcomes of knowledge.

**Table.** Summary Statistics by Sample

Characteristic	No. (%)	
	National sample (n = 1515)	Opioid dependence sample (n = 562)^a^
Gender		
Men	739 (48.8)	158 (28.1)
Women	770 (50.8)	404 (71.9)
Self-identify	6 (0.4)	0
Education (ages ≥25 y)		
High school or less	360 (23.8)	188 (33.5)
Some college	310 (20.5)	171 (30.4)
Advanced associate degree or trade school	238 (15.7)	125 (22.2)
Bachelors degree	407 (26.9)	59 (10.5)
Graduate degree	200 (13.2)	19 (3.4)
Race^b^		
American Indian or Alaska Native	20 (1.3)	13 (2.3)
Asian American or Pacific Islander	101 (6.7)	10 (1.8)
Black	215 (14.2)	17 (3.0)
Other race	68 (4.5)	13 (2.3)
White	1087 (71.7)	494 (87.9)
Multiracial	24 (1.6)	15 (2.7)
Ethnicity		
Hispanic	256 (16.9)	65 (11.6)
Age group, y		
18-24	186 (12.3)	8 (1.4)
25-34	242 (16.0)	105 (18.7)
35-44	327 (21.6)	238 (42.3)
45-54	280 (18.5)	136 (24.2)
55-64	281 (18.5)	57 (10.1)
65-74	139 (9.2)	16 (2.8)
75-84	60 (4.0)	2 (0.4)
Political party		
Republican	553 (36.5)	220 (39.1)
Democratic	628 (41.5)	171 (30.4)
Other	334 (22.0)	171 (30.4)
Region		
Northeast	276 (18.2)	100 (17.8)
Midwest	328 (21.7)	118 (21.0)
South	560 (37.0)	229 (40.7)
West	351 (23.2)	115 (20.5)
Opioid dependence	50 (3.3)	562 (100.0)

^a^ Includes 50 respondents from the national sample who self-reported opioid dependence.

^b^ Survey respondents could identify race as American Indian or Alaska Native, Asian, Asian American or Pacific Islander, Black, White, or other. Individuals who chose other could write in their race. In most cases, respondents wrote Hispanic or Latino.

The sample reporting prior or current opioid dependence included 512 respondents from an oversample and 50 national sample respondents (3.3%) who also reported prior or current opioid dependence. Together a total of 562 respondents were included in the opioid dependence sample, 404 (71.9%) of whom identified as women. While prescription opioid use is higher among women than men, the opposite is true of dependence.^33^ Relative to respondents in the 2022 National Survey on Drug and Health (NSDUH) who have opioid use disorder based on the *Diagnostic and Statistical Manual of Mental Disorders* (Fifth Edition) (*DSM-5*), the opioid dependence sample was also disproportionately between the ages of 35 to 49 years (329 [58.5%]), identified as non-Hispanic White (452 [80.4%]), and had some college but no degree (167 respondents [52.6%] among those aged 26 years and older) (eTable 2 in Supplement 1). Demographics for the unrestricted survey samples, which included respondents who failed the attention check or did not otherwise complete the survey, were similar to those for the analytic samples (eTable 3 in Supplement 1).

In the national sample, 169 respondents (11.2%) reported knowing someone very likely to have an opioid overdose and 71 (4.7%) reported that they themselves were very likely to have an opioid overdose (eTable 4 in Supplement 1). Over the past 12 months, 208 respondents (13.7%) reported using prescription opioids in a way not directed by a doctor or using nonprescription opioids such as heroin or illicitly made fentanyl, and 114 (7.5%) reported using illicit fentanyl specifically. Among those reporting opioid dependence, 271 (48.2%) reported knowing someone very likely to have an opioid overdose but only 42 (7.5%) reported that they themselves were very likely to have an opioid overdose. Over the past 12 months, 359 individuals (63.9%) indicating opioid dependence reported using prescription opioids in a way not directed by a doctor or using nonprescription opioids; 242 (43.1%) reported illicit fentanyl specifically.

### Main Findings

**Figure 1.**
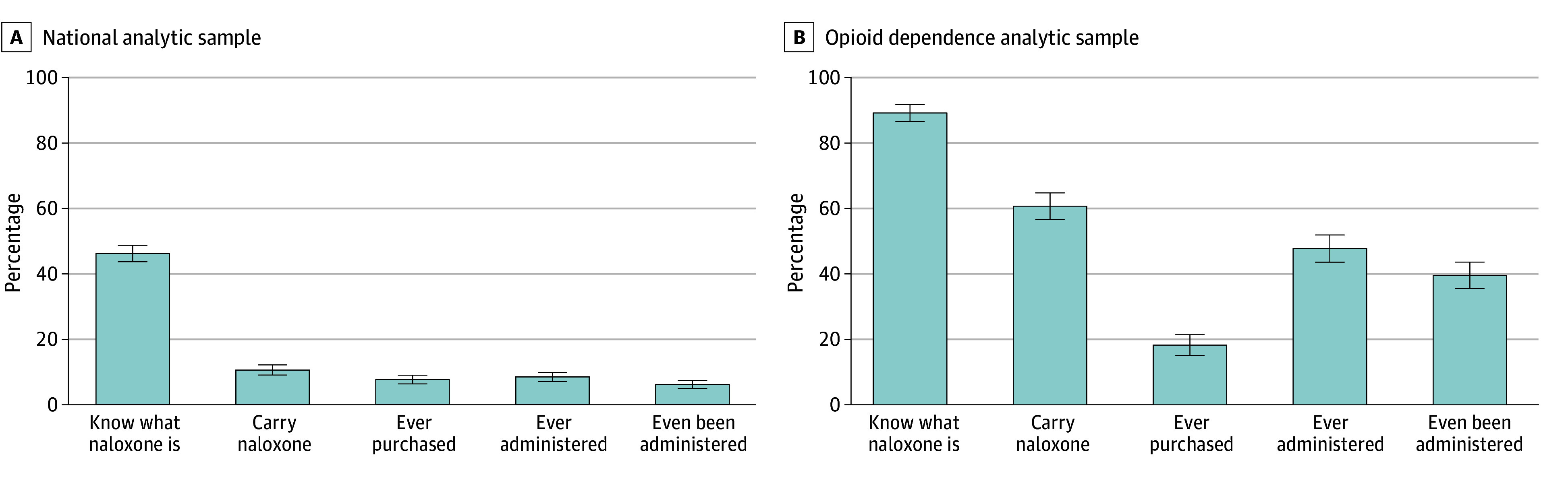
Naloxone Knowledge, Carrying, Purchase, and Administration in a National Sample and Among Opioid Dependence Respondents A, The national sample includes 1515 respondents; B, the sample reporting opioid dependence includes 562 respondents. Whiskers indicate 95% CIs. People coded as “carry naloxone” if they self-reported that they currently carry naloxone “occasionally” or “most or all of the time.”

Of the 1515 respondents in the national sample, only 46.2% (95% CI, 43.7%-48.7%) said they had heard of naloxone and correctly identified its use, 10.6% (95% CI, 9.0%-12.1%) reported currently carrying naloxone, 7.7% (95% CI, 6.3%-9.0%) had ever purchased naloxone, 8.5% (95% CI, 7.1%-9.9%) reported having administered naloxone to someone else, 6.1% (95% CI, 4.9%-7.4%) having been administered the medication (Figure 1A). Knowledge, carry, and administration rates were higher for the opioid dependence group. Of the 562 respondents reporting opioid dependence, 89.0% (95% CI, 86.4%-91.6%) had heard of naloxone and correctly identified its use, 60.5% (95% CI, 56.4%-64.6%) reported currently carrying naloxone, 18.0% (95% CI, 14.8%-21.2%) had ever purchased naloxone, 47.5% (95% CI, 43.4%-51.7%) reported administering naloxone to someone else, and 39.3% (95% CI, 35.3%-43.4%) having been administered the medication (Figure 1B).

### Knowledge by Type of Opioid Exposure

**Figure 2.**
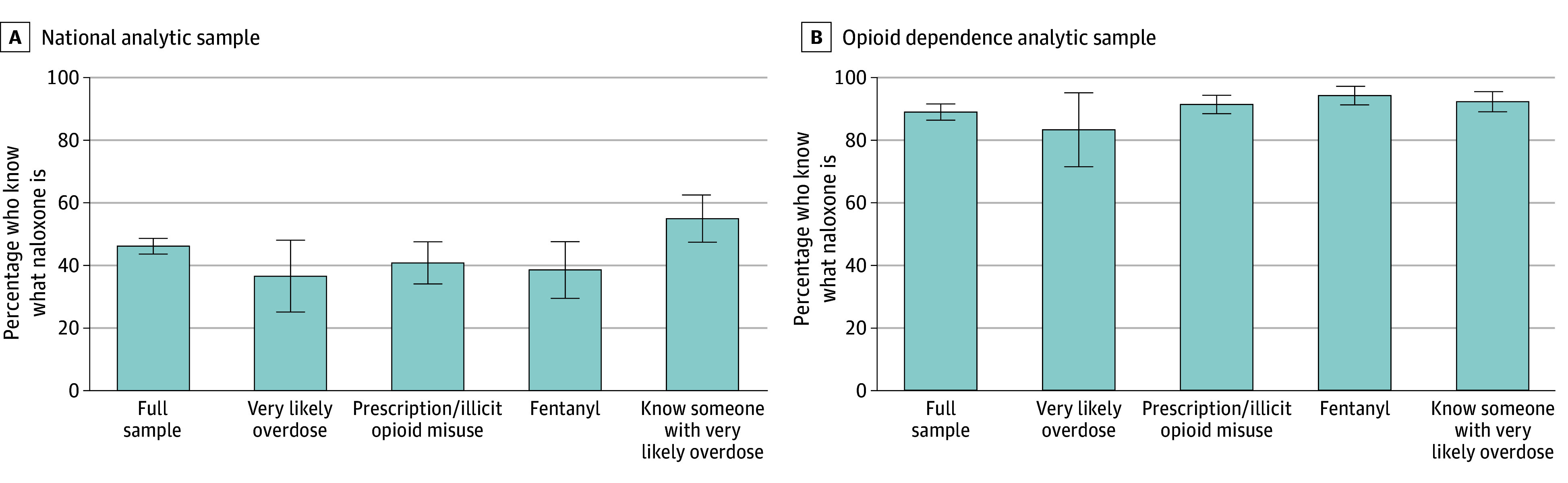
Naloxone Knowledge by Subgroups of Types of Opioid Exposure Whiskers indicate 95% CIs. A, The sample size for each column is 1515 respondents in the full sample, 71 reporting they were very likely to overdose from opioid use, 208 reporting misuse of prescription opioids or use of illicit opioids with the past 12 months, 114 reporting illicitly made fentanyl use within the past 12 months, and 169 reporting that they know someone who is very likely to overdose from opioid use. B, The sample sizes for these measures are 562 in the full sample, 42 very likely to overdose, 359 reporting opioid misuse, 242 reporting fentanyl use, and 271 reporting an acquaintance likely to overdose.

Knowledge of naloxone varied modestly across different dimensions of opioid exposure (Figure 2). Among those who said they were very likely to overdose, 36.6% (95% CI, 25.2%-48.1%) knew what naloxone was. Knowledge among those using prescription opioids nonmedically or using illicit opioids within the past 12 months was similar, with 40.9% (95% CI, 34.1%-47.6%) correctly identifying naloxone. Among those reporting illicit fentanyl use in the past 12 months, 38.6% (95% CI, 29.5%-47.7%) correctly identified naloxone’s purpose. In contrast, 55.0% (95% CI, 47.5%-62.6%) of those who said they knew someone very likely to overdose correctly identified the medication’s purpose.

Knowledge of naloxone was much higher among the sample reporting current or prior opioid dependence. Among those reporting opioid dependence, the percentage who correctly identified naloxone’s purpose was 83.3% (95% CI, 71.6%-95.1%) for those who said they were very likely to overdose. Among those with opioid dependence and self-reported prescription or illicit opioid misuse within the past 12 months, the proportion was 91.4% (95% CI, 88.5%-94.3%), increasing to 94.2% (95% CI, 91.3%-97.2%) for those reporting illicitly made fentanyl use specifically. For those who said that they know someone very likely to overdose, the proportion was 92.3% (95% CI, 89.1%-95.5%).

### Carrying by Type of Opioid Exposure

**Figure 3.**
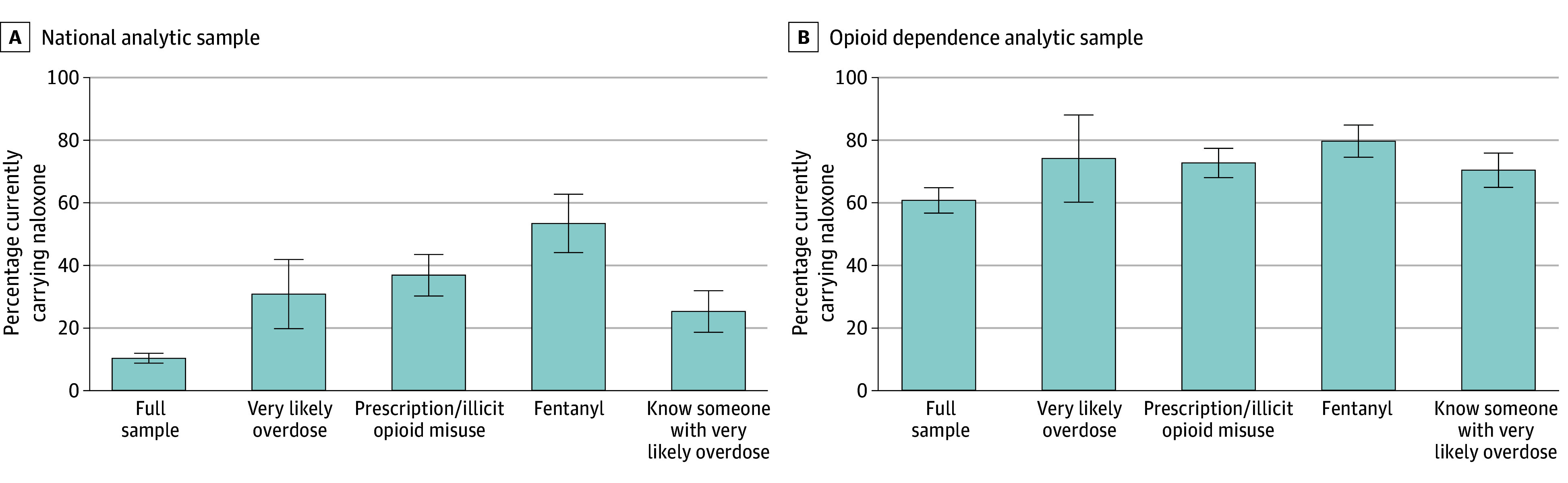
Proportion Currently Carrying Naloxone by Subgroup Currently carrying naloxone is defined as the respondent reporting that they currently carry naloxone “occasionally” or “most or all of the time.” Whiskers indicate 95% CIs. A, The sample size for each column is 1515 respondents in the full sample, 71 reporting they were very likely to overdose from opioid use, 208 reporting misuse of prescription opioids or use of illicit opioids with the past 12 months, 114 reporting illicitly made fentanyl use within the past 12 months, and 169 reporting that they know someone who is very likely to overdose from opioid use. B, The sample sizes for these measures are 562 in the full sample, 42 very likely to overdose, 359 reporting opioid misuse, 242 reporting fentanyl use, and 271 reporting an acquaintance likely to overdose.

The proportion currently carrying naloxone increased with opioid exposure and risk (Figure 3). While less than 11% of the overall sample carried naloxone, 31.0% (95% CI, 20.0%-42.0%) of those who said they were very likely to overdose currently carried naloxone. Proportions currently carrying naloxone were 37.0% (95% CI, 30.4%-43.6%) among those either using prescription opioids in a way not directed by a doctor or using illicit opioids, rising to 53.5% (95% CI, 44.2%-62.8%) among those reporting illicit fentanyl use within the past 12 months. Among those responding that they knew someone very likely to overdose from opioid use, 25.4% (95% CI, 18.8%-32.1%) reported currently carrying naloxone.

Naloxone carrying was higher among those reporting opioid dependence: 73.8% (95% CI, 59.9%-87.7%) among those who also said they were very likely to overdose, 72.4% (95% CI, 67.8%-77.1%) among those reporting misuse of prescription or illicit opioids in the past 12 months, and 79.3% (95% CI, 74.2%-84.5%) among those reporting illicit fentanyl use in the past 12 months. Carry rates were 70.1% (95% CI, 64.6%-75.6%) among those who said they knew someone very likely to overdose.

The proportion purchasing naloxone among those who had ever carried naloxone was low (eFigure 2 in Supplement 1). Among those who ever carried naloxone, only 42.4% (95% CI, 36.3%-48.5%) of the national sample and 22.6% (95% CI, 18.6%-26.5%) of the opioid dependence sample reported ever purchasing naloxone.

## Discussion

Increasing access to naloxone, a highly effective opioid overdose reversal medication, is a primary policy response to opioid overdose deaths in the US,^5^ even as polysubstance overdoses become increasingly common.^34^ Available over the counter, through health center giveaways and through vending machines in some communities, naloxone is seemingly ubiquitous. Yet, to our knowledge, no national data track whether people know what naloxone is, carry the medication, or use it, making it difficult to measure progress on naloxone access. This study aimed to provide timely estimates of naloxone knowledge, carrying, and administration nationally and among an oversample of individuals reporting opioid dependence.

The survey demonstrated that naloxone knowledge and carrying were higher among those with greatest potential need, suggesting individuals respond to heightened risk. About 46% of adults in the national sample correctly identified naloxone as a medication to reverse an opioid overdose, rising to 89% among those reporting opioid dependence. Likewise, just under 11% of respondents in the national sample reported carrying naloxone either “occasionally” or “most or all of the time,” while nearly 61% of those reporting opioid dependence currently carry naloxone.

Carrying increased with self-assessed likelihood of overdose or knowing someone likely to overdose. About 25% of respondents who reported knowing someone very likely to overdose and 31% who reported that they themselves were very likely to overdose also reported currently carrying naloxone. Among those reporting opioid dependence, about 74% of those identifying as very likely to overdose reported that they currently carry naloxone. This percentage is remarkably similar to the 71% reporting current possession in a recent survey of adults at high risk of overdose in 3 states.^35^

Notably, there were sizeable gaps between the percentages of people reporting ever carrying and ever purchasing naloxone. In the national sample, about 42.4% of those who reported ever carrying also reported ever purchasing naloxone. These differences were even more striking among the sample reporting opioid dependence: only 22% of those who reported ever carrying naloxone also reported ever purchasing naloxone. These data suggested that pharmacy sales and insurance claims understate the proportion of people who possess and carry naloxone, particularly among those at highest need. Alternative means of capturing naloxone access, such as rapid online surveys, are needed to monitor naloxone access.

Finally, the survey provides needed data on naloxone use. Administration was high among the sample reporting opioid dependence, with 47.5% reporting administering naloxone to someone else and 39.3% reporting having naloxone administered to them. While we relied on respondents who passed the attention check for the main analysis, the study patterns were similar when including those who did not (eFigures 3 through 5 in Supplement 1).

### Limitations

This study has several limitations. First, the survey was conducted online. Respondents needed internet access and, implicitly, some comfort navigating a digital survey. It is unclear how excluding people without internet access affected our findings. Perhaps relatedly, our sample had higher educational attainment than the general population. Together these factors suggest we might overstate naloxone knowledge and understate opioid misuse and naloxone carrying and administration. However, to the extent that internet access correlates negatively with opioid misuse, we might understate not only naloxone carrying and administration but also knowledge. Second, survey data were self-reported. Although we required respondents to correctly identify the use of naloxone for the knowledge question, all answers are subject to reporting bias. Nonetheless, the use of an attention check to screen out respondents filling out the survey inattentively and internal consistency checks of survey responses (eAppendix 5, eFigure 6 in Supplement 1) gave us some confidence in the data quality. Third, the survey, like the NSDUH, excluded institutionalized individuals. This exclusion leads us to underrepresent individuals with substance use disorder and from minority ethnic and racial populations, who are overrepresented in the carceral settings.^36,37^ Fourth, the self-identified opioid dependence sample was unrepresentative of the population with opioid dependence. Given the difficulty of surveying this population, information from a convenience sample is valuable. In particular, survey responses from the sample reporting opioid dependence may be informative about those with concerns about their opioid use. Fifth, due to the cross-sectional nature of the survey design, we cannot identify the relationship between naloxone policy and access. These data should be understood as a first step to understanding naloxone access at a point in time and, combined with future data collection, may be useful in understanding policy impacts.

## Conclusions

This online survey provided timely data on naloxone knowledge, carrying and administration in a national sample of US adults and an oversample of adults reporting opioid dependence. These data, which should be validated in other samples, suggest naloxone knowledge and access are high, particularly among high-risk groups. Increasing carrying among individuals who are not themselves high-risk but know people who are may be a useful avenue for further improving access and reducing the risk of opioid overdose death.

## Notes

Supplement 1. **eAppendix 1.** Introductory Text to the Survey **eAppendix 2.** Comparing Survey Samples to National Survey Data **eTable 1.** Demographic and Geographic Characteristics of the Nationally Representative and Opioid Dependent Survey Samples with Rates from the May 2024 Current Population Study **eTable 2.** Demographic Characteristics of the Opioid Dependence Sample and the Sample with Opioid Use Disorder in the 2022 National Survey on Drug Use and Health (NSDUH) **eAppendix 3.** Sample Restrictions **eFigure 1.** Flow Diagram of Analytic Samples **eTable 3.** Comparing Samples with and without Attention Check and Completeness Restrictions **eTable 4.** Measures of Opioid Exposure Across Samples **eFigure 2.** Rates of Ever Purchasing Naloxone Among Respondents Reporting Ever Carrying Naloxone, Presented by Sample **eFigure 3.** Naloxone Knowledge, Carrying, Purchase, and Administration in a National Sample and Among Opioid Dependence Respondents **eFigure 4.** Naloxone Knowledge by Subgroups of Opioid Exposure **eFigure 5.** Proportion Currently Carrying Naloxone by Subgroup **eAppendix 4.** Internal Consistency of Responses **eFigure 6.** Personal Impacts of Opioid Use Among Respondents With No Prescription or Illicit Opioid Use Within Past 12 Months **eFigure 7.** Self-Reported Likelihood of Overdosing From Opioid Use for Respondents With No Prescription or Illicit Opioid Use Within Past 12 Months and No Personal Impact from Opioid Use **eAppendix 5.** Full Survey

Supplement 2.
Data Sharing Statement
