# Supplementary material for: Naloxone Knowledge, Carrying, Purchase, and Use
Source: JAMA Netw Open. 2025 Mar 3;8(3):e2462698. doi: 10.1001/jamanetworkopen.2024.62698 (PMC11877168; doi:10.1001/jamanetworkopen.2024.62698)
Supplement: Supplement 1. — eAppendix 1. Introductory Text to the Survey eAppendix 2. Comparing Survey Samples to National Survey Data eTable 1. Demographic and Geographic Characteristics of the Nationally Representative and Opioid Dependent Survey Samples with Rates from the May 2024 Current Population Study eTable 2. Demographic Characteristics of the Opioid Dependence Sample and the Sample with Opioid Use Disorder in the 2022 National Survey on Drug Use and Health (NSDUH) eAppendix 3. Sample Restrictions eFigure 1. Flow Diagram of Analytic Samples eTable 3. Comparing Samples with and without Attention Check and Completeness Restrictions eTable 4. Measures of Opioid Exposure Across Samples eFigure 2. Rates of Ever Purchasing Naloxone Among Respondents Reporting Ever Carrying Naloxone, Presented by Sample eFigure 3. Naloxone Knowledge, Carrying, Purchase, and Administration in a National Sample and Among Opioid Dependence Respondents eFigure 4. Naloxone Knowledge by Subgroups of Opioid Exposure eFigure 5. Proportion Currently Carrying Naloxone by Subgroup eAppendix 4. Internal Consistency of Responses eFigure 6. Personal Impacts of Opioid Use Among Respondents With No Prescription or Illicit Opioid Use Within Past 12 Months eFigure 7. Self-Reported Likelihood of Overdosing From Opioid Use for Respondents With No Prescription or Illicit Opioid Use Within Past 12 Months and No Personal Impact from Opioid Use eAppendix 5. Full Survey [file jamanetwopen-e2462698-s001.pdf]

# Supplemental Online Content

Jacobson M, Powell D. Naloxone knowledge, carrying, and use among US adults. *JAMA Netw Open.* 2025;8(3):e2462698. doi:10.1001/jamanetworkopen.2024.62698

**eAppendix 1.** Introductory Text to the Survey

**eAppendix 2.** Comparing Survey Samples to National Survey Data

**eTable 1.** Demographic and Geographic Characteristics of the Nationally Representative and Opioid Dependent Survey Samples with Rates from the May 2024 Current Population Study

**eTable 2.** Demographic Characteristics of the Opioid Dependence Sample and the Sample with Opioid Use Disorder in the 2022 National Survey on Drug Use and Health (NSDUH)

**eAppendix 3.** Sample Restrictions

**eTable 3.** Comparing Samples with and without Attention Check and Completeness Restrictions

**eTable 4.** Measures of Opioid Exposure Across Samples

**eFigure 2.** Rates of Ever Purchasing Naloxone Among Respondents Reporting Ever Carrying Naloxone, Presented by Sample

**eFigure 3.** Naloxone Knowledge, Carrying, Purchase, and Administration in a National Sample and Among Opioid Dependence Respondents

**eFigure 4.** Naloxone Knowledge by Subgroups of Opioid Exposure

**eFigure 5.** Proportion Currently Carrying Naloxone by Subgroup

**eAppendix 4.** Internal Consistency of Responses

**eFigure 6.** Personal Impacts of Opioid Use Among Respondents With No Prescription or Illicit Opioid Use Within Past 12 Months

**eFigure 7.** Self-Reported Likelihood of Overdosing From Opioid Use for Respondents With No Prescription or Illicit Opioid Use Within Past 12 Months and No Personal Impact from Opioid Use

**eAppendix 5.** Survey

This supplemental material has been provided by the authors to give readers additional information about their work.

© 2025 Jacobson M et al. *JAMA Network Open*.

eAppendix 1. Introductory Text to the Survey

The first screen seen by participants included the following text:

*This survey asks about your knowledge of and personal experiences with substance use in the United States. The survey is for a research study conducted by RAND and the University of Southern California. It includes sensitive questions about prior substance use. Your responses are confidential and the researchers receiving the data will not have access to any identifying information. Please do not include any personally identifying information in any of your responses. You can stop the survey at any time. We know that this information is personal, but your answers are important to our understanding of substance use in the US. This survey should take 10 minutes of your time.*

*If the topics in this survey bring up issues that you would like to discuss with someone. Please call the Substance Abuse and Mental Health Services (SAMHSA) helpline: 1-800-662-3457.*

*This study was approved by the RAND Human Subjects Protection Committee and has also been reviewed by the USC Institutional Review Board (IRB). The IRB is a research review board that reviews and monitors research studies to protect the rights and welfare of research participants. Contact the IRB if you have questions about your rights as a research participant or have complaints about the research. You can contact RAND's Human Subjects Protection Committee toll-free at (866) 697-5620 or by emailing hspcinfo@rand.org. When you contact the RAND Committee, please reference Study #2023-N0309. You can also contact USC's IRB at (323) 442-0114 or by email at hrpp@usc.edu. When you contact USC's IRB, please reference Study UP -24-00279.*

Participants were then asked to check a box signifying that they wanted to proceed:

*I've read the information above and agree to proceed with the survey*

© 2025 Jacobson M et al. *JAMA Network Open*.

eAppendix 2. Comparing Survey Samples to National Survey Data

S.2.1 The Nationally Survey Sample Compared to the May 2024 Current Population Survey (CPS)

Our sample included 1,515 completed survey responses. The first questions in the survey asked for demographic information. In this supplemental section, we compare the demographics of our sample to the May 2024 Current Population Survey (CPS), the most recent monthly CPS available. The CPS is the primary source of monthly labor force statistics in the United States and provides detailed information on the demographic characteristics of US residents. eTable 1 provides the demographic characteristics for the national sample (column 1) and for the population aged 18 and over in the May 2024 CPS (column 2).

The national sample is similar based on gender to the CPS, with about 49% in each sample identifying as male. We permitted respondents in our survey to select male or female or to self-identify. The CPS does not provide this latter option.

The survey also asked about the respondent's race. Respondents could select one or more options regarding their race. Given the small sample size, we grouped respondents into "Black," "White," and "Other," in which the last category includes all other responses and anyone who selected more than one race. Our national sample had higher shares of individuals identifying as Black or "Other Race" compared to the CPS and a lower share identifying as White.

The survey also asked about Hispanic ethnicity, and we found that our sample and the CPS had similar proportions of individuals identifying as Hispanic. The age distribution was also similar, although our sample had a smaller share of respondents aged 75+ and higher shares at younger parts of the age distribution. The largest difference between our sample and the CPS related to educational attainment. Our sample was less likely to have never attended any college (i.e., high school degree or less). The geographical distribution of survey respondents, based on Census region, was similar to that of the CPS.

S.2.2 The Opioid Dependence Sample Compared to the 2022 National Survey on Drug Use and Health (NSDUH) Sample with Opioid Use Disorder

The opioid dependence sample included a total of 562 completed survey responses, 512 from the targeted sample previously reporting opioid dependence and 50 from the national sample who indicated opioid dependence. Opioid dependence was just one of nine conditions that respondents could select as health issues they "are currently suffering from or have ever suffered from." The order of conditions, which included alcohol dependence, asthma, cancer, cardiovascular disease, chronic kidney disease, chronic liver disease, depression, and diabetes, was randomized across respondents.

We compared the demographics of the sample for respondents reporting opioid dependence to the 2022 National Survey on Drug Use and Health (NSDUH), the most recent NSDUH available. The NSDUH is the primary source of nationally representative data on tobacco, alcohol, and drug use among the civilian, noninstitutionalized population aged 12 or older in the United

© 2025 Jacobson M et al. *JAMA Network Open*.

States. eTable 2 provides the demographic characteristics for the sample reporting opioid dependence (column 1) and the population aged 18 and over with Opioid Use Disorder (OUD) in the NSDUH (column 2). OUD was determined using the NSDUH-constructed DSM-5 based substance use disorder (SUD) variables and thus is not directly comparable to self-reported opioid dependence. The NSDUH only includes broad age categories, so we report age group shares in our samples according to those same categorizations. We also used the same race categorization as the NSDUH for eTable 2. For education, we report rates for the aged 26+ population (it is not possible to identify the 25+ population).

Females and White respondents are over-represented in the sample reporting opioid dependence while males and non-Hispanic Black, non-Hispanic other race, and Hispanic respondents are underrepresented. The opioid dependence sample is also disproportionately between the ages of 26 to 34 and 35 to 49, with small proportions in the youngest (ages 18-25) and oldest (ages 50-64 and 65+) age groups. Our sample was also less likely to have never attended any college.

© 2025 Jacobson M et al. *JAMA Network Open*.

eTable 1. Demographic and Geographic Characteristics of the Nationally Representative and Opioid Dependent Survey Samples with Rates from the May 2024 Current Population Study

| | | Nat Rep | CPS |
|---|---|---|---|
| **Gender** | Male | 48.78% | 48.74% |
| | Female | 50.83% | 51.26% |
| | Self-Identify | 0.40% | |
| **Race** | Black | 14.19% | 12.96% |
| | White | 71.75% | 76.54% |
| | Other Race | 14.06% | 10.50% |
| **Ethnicity** | Hispanic | 16.90% | 17.95% |
| **Age** | 18-24 | 12.28% | 11.53% |
| | 25-34 | 15.97% | 17.15% |
| | 35-44 | 21.58% | 17.02% |
| | 45-54 | 18.48% | 15.45% |
| | 55-64 | 18.55% | 15.92% |
| | 65-74 | 9.17% | 13.48% |
| | 75+ | 3.96% | 9.45% |
| **Education** | No College | 23.76% | 38.15% |
| | Some College | 36.17% | 15.48% |
| | College Degree | 40.07% | 36.09% |
| **Region** | Midwest | 21.65% | 20.43% |
| | Northeast | 18.22% | 17.33% |
| | South | 36.96% | 38.64% |
| | West | 23.17% | 23.59% |
| **Sample Size** | | 1,515 | 79,350 |

*Notes: Nat Rep = Nationally-Representative sample. In the last column, we used the May 2024 Current Population Survey and their population-weights to construct comparable rates. Educational attainment is summarized for the population aged 25+. "Other Race" includes all respondents not identified as White or Black, including those selecting more than one race.*

© 2025 Jacobson M et al. *JAMA Network Open*.

eTable 2. Demographic Characteristics of the Opioid Dependence Sample and the Sample with Opioid Use Disorder in the 2022 National Survey on Drug Use and Health (NSDUH)

| | | Opioid Dependence | NSDUH |
|---|---|---|---|
| **Gender** | Male | 28.11% | 50.08% |
| | Female | 71.89% | 49.92% |
| | Self-Identify | 0.00% | |
| **Race** | Non-Hispanic Black | 2.67% | 15.43% |
| | Non-Hispanic White | 80.43% | 58.99% |
| | Non-Hispanic, Other Race | 5.34% | 9.16% |
| **Ethnicity** | Hispanic | 11.57% | 16.42% |
| **Age** | 18-25 | 1.96% | 6.91% |
| | 26-34 | 18.15% | 13.54% |
| | 35-49 | 58.54% | 29.41% |
| | 50-64 | 18.15% | 32.47% |
| | 65+ | 3.20% | 17.68% |
| **Education** | No College | 33.21% | 48.25% |
| (Ages 26+) | Some College | 52.63% | 39.00% |
| | College Degree | 14.16% | 12.75% |
| **Sample Size** | | 562 | 905 |

*Notes: The opioid dependence sample includes 50 respondents from the nationally representative sample who answered that they currently or previously had opioid dependence. In the last column, we used the 2022 NSDUH (Ages 18+). OUD was determined using the NSDUH-constructed DSM-5 based SUD variables, which is not directly comparable to the self-reported measure of dependence used in our survey. Race and age categorizations differ relative to eTable 1 because of differences in the information available in the NSDUH. Educational attainment is summarized for the population ages 26+.*

© 2025 Jacobson M et al. *JAMA Network Open*.

## eAppendix 3. Sample Restrictions

### Attention Check and Incomplete Surveys

Both analytic samples excluded respondents who failed an attention check (n=746 nationally; 147 opioid dependence) or who did not complete the survey (n=29 nationally; 17 opioid dependence). An attention check is a commonly used approach to screen out individuals who are mindlessly answering a survey. The flow diagrams in eFigure 1 (Panels A and B) demonstrate the sample composition.

### Comparison of Summary Statistics

In this section, we consider whether these exclusions may have unnecessarily skewed our sample. In eTable 3, we compute the summary statistics from Table 1 but without the attention check or completeness requirements for the full national sample (col 1) and the sample reporting opioid dependence (col 3). We also reproduce the summary statistics for the analytic samples, national (col 2) and those reporting opioid dependence (col 4). The gender distributions were quite similar with and without restrictions. The analytic sample was, however, slightly more educated, with 23.8% having a high school degree or less compared to 29% of the national sample without the attention check or completeness requirement. Individuals in the national analytic sample were also more likely to identify as White, 71.8% versus 62.7% compared to unrestricted national sample and less likely to identify as multi-racial, 1.58% versus 8.3%. Hispanic ethnicity, age distribution and party affiliation were similar. Respondents in the national analytic sample were more likely to be from the West (23.2% versus 19.9%) and less likely to be from the Northeast (18.2% versus 23.1%). For the sample reporting opioid dependence, the main divergence based on the sample restriction was for education, with 33.5% of the analytic sample having a high school degree or less compared to 36.2% in the sample without the attention check or completeness requirement.

© 2025 Jacobson M et al. *JAMA Network Open*.

eFigure 1. Flow Diagram of Analytic Samples

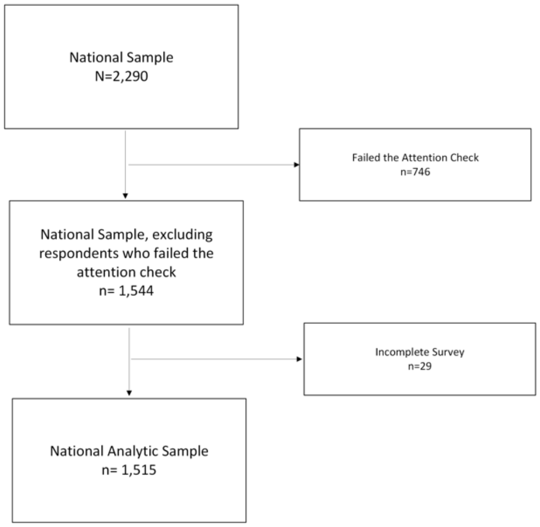


Panel A. National Sample

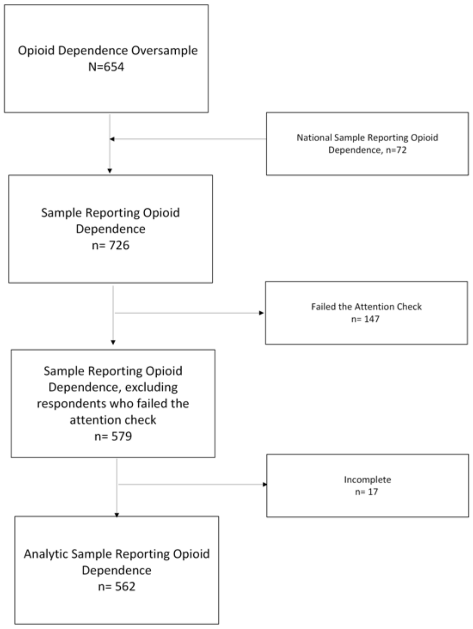


Panel B. Respondents Reporting Opioid Dependence

© 2025 Jacobson M et al. *JAMA Network Open*.

eTable 3. Comparing Samples with and without Attention Check and Completeness Restrictions

| Summary Statistics by Sample, No. (%)<br>Characteristic | Full National Sample [a] (n=2,290) | National Sample (n=1,515) | Full Opioid Dependence Sample[b] (n=726) | Opioid Dependence Sample[c] (n=562) |
|---|---|---|---|---|
| **Gender** | | | | |
| Male | 1091 (49.8) | 739 (48.8) | 219 (30.2) | 158 (28.1) |
| Female | 1094 (49.9) | 770 (50.8) | 507 (69.8) | 404 (71.9) |
| Self-identify | 8 (0.37) | 6 (0.40) | | |
| **Education** (ages 25+) | | | | |
| HS or less | 620 (29.0) | 360 (23.8) | 263 (36.2) | 188 (33.5) |
| Some college | 413 (19.3) | 310 (20.5) | 213 (29.3) | 171 (30.4) |
| AA degree or trade school | 327 (15.3) | 238 (15.7) | 153 (21.1) | 125 (22.2) |
| BA degree | 513 (24.0) | 407 (26.9) | 75 (10.3) | 59 (10.5) |
| Graduate degree | 264 (12.4) | 200 (13.2) | 22 (3.03) | 19 (3.38) |
| **Race** [d] | | | | |
| AI/AN | 35 (1.53) | 20 (1.32) | 21 (2.89) | 13 (2.31) |
| AA/PI | 140 (6.11) | 101 (6.67) | 13 (1.79) | 10 (1.78) |
| Black | 388 (16.9) | 215 (14.2) | 26 (3.58) | 17 (3.02) |
| White | 1436 (62.7) | 1087 (71.8) | 633 (87.2) | 494 (87.9) |
| Other Race | 101 (4.41) | 68 (4.49) | 15 (2.07) | 13 (2.31) |
| Multi-racial | 190 (8.3) | 24 (1.58) | 18 (2.48) | 15 (2.67) |
| **Ethnicity** | | | | |
| Hispanic | 377 (17.7) | 256 (16.9) | 81 (11.2) | 65 (11.6) |
| **Age group** [e] | | | | |
| 18-24 | 281 (13.1) | 186 (12.3) | 15 (2.07) | 8 (1.42) |
| 25-34 | 369 (17.2) | 242 (16.0) | 148 (20.4) | 105 (18.7) |
| 35-44 | 474 (22.1) | 327 (21.6) | 290 (40.0) | 238 (42.4) |
| 45-54 | 401 (18.7) | 280 (18.5) | 177 (24.4) | 136 (24.2) |
| 55-64 | 379 (17.7) | 281 (18.6) | 74 (10.2) | 57 (10.1) |
| 65-74 | 166 (7.75) | 13 (9.17) | 19 (2.62) | 16 (2.85) |
| 75-84 | 73 (3.41) | 60 (4.0) | 3 (0.41) | 2 (0.36) |
| **Political Party** | | | | |
| Republican | 746 (35.0) | 553 (36.5) | 284 (39.1) | 220 (39.2) |
| Democratic | 920 (43.1) | 628 (41.5) | 215 (29.6) | 171 (30.4) |
| Other | 467 (21.9) | 334 (22.1) | 227 (31.3) | 171 (30.4) |
| **Region** | | | | |
| Northeast | 528 (23.1) | 276 (18.2) | 128 (17.6) | 100 (17.8) |
| Midwest | 453 (19.8) | 328 (21.7) | 160 (22.0) | 118 (21.0) |

© 2025 Jacobson M et al. *JAMA Network Open*.

| | | | | |
|---|---|---|---|---|
| South | 854 (37.3) | 560 (37.0) | 297 (40.9) | 229 (40.8) |
| West | 455 (19.9) | 351 (23.2) | 141 (19.4) | 115 (20.5) |
| **Failed Attention Check** | 746 (32.6) | | 147 (20.3) | |
| **Incomplete only** | 29 (1.27) | | 17 (2.34) | |
| **Opioid Dependence** | 72 (3.14) | 50 (3.3) | 726 (100) | 562 (100) |

[a] Sub-group sample sizes do not always sum to the overall total (n=2,290) due to missing data. E.g., the sample size across gender categories sum to 2,193.
[b] Includes 72 respondents from the full national sample who self-reported opioid dependence.
[c] Includes 50 respondents from the national analytic sample who self-reported opioid dependence.
[d] Survey respondents could identify race as American Indian or Alaska Native =AI/AN, Black, Asian, Asian American or Pacific Islander=AA/PI, White, or Other. Individuals who chose "Other" could write-in their race. In most cases, respondents wrote "Hispanic" or "Latino." [e] Age was measured in years.

© 2025 Jacobson M et al. *JAMA Network Open*.

eTable 4. Measures of Opioid Exposure Across Samples

| Characteristic | No. (%) | |
|---|---|---|
| | Nationally Representative Sample (n=1,515) | Opioid Dependent Respondents (n=562) |
| Know someone “very likely” to overdose | 169 (11.2) | 271 (48.2) |
| “Very likely” to overdose | 71 (4.7) | 42 (7.5) |
| Misused prescription opioids or used non-prescription opioids in the past 12 months | 208 (13.7) | 359 (63.9) |
| Used illicit fentanyl in the past 12 months | 114 (7.5) | 242 (43.1) |

© 2025 Jacobson M et al. *JAMA Network Open*.

eFigure 2. Rates of Ever Purchasing Naloxone Among Respondents Reporting Ever Carrying Naloxone, Presented by Sample

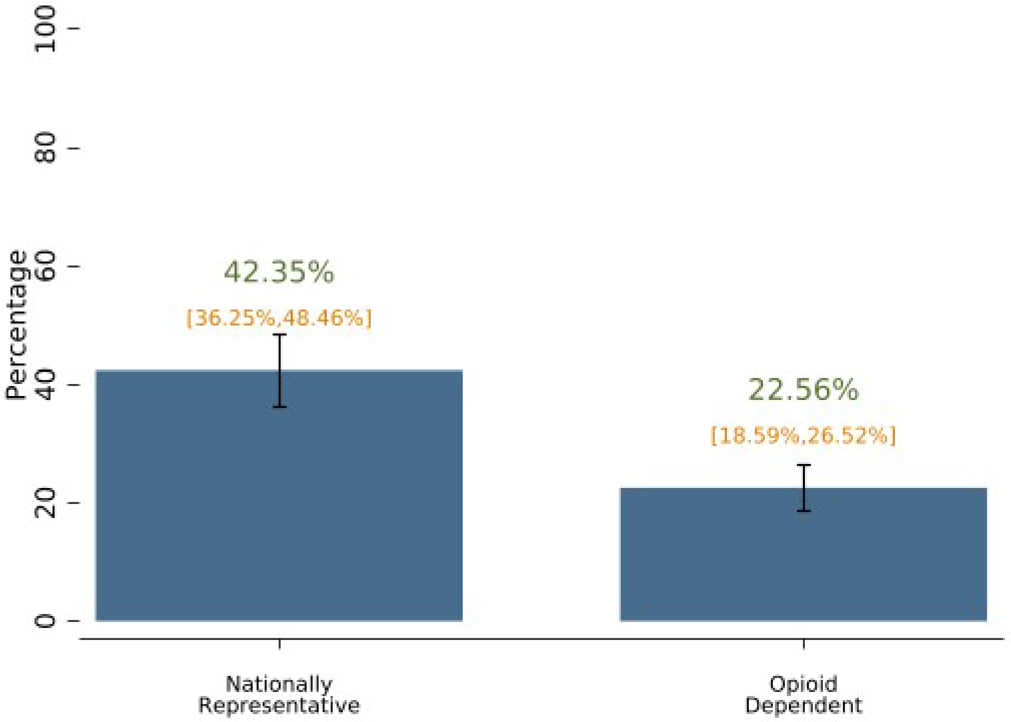


*Notes: The first bar represents the percentage of respondents in the nationally-representative sample who have ever purchased naloxone among those who have ever carried naloxone (N=255). The second bar represents the percentage of respondents who have ever purchased naloxone among those reporting opioid dependence who have ever carried naloxone (N=430).*

© 2025 Jacobson M et al. *JAMA Network Open*.

eFigure 3. Naloxone Knowledge, Carrying, Purchase, and Administration in a National Sample and Among Opioid Dependence Respondents

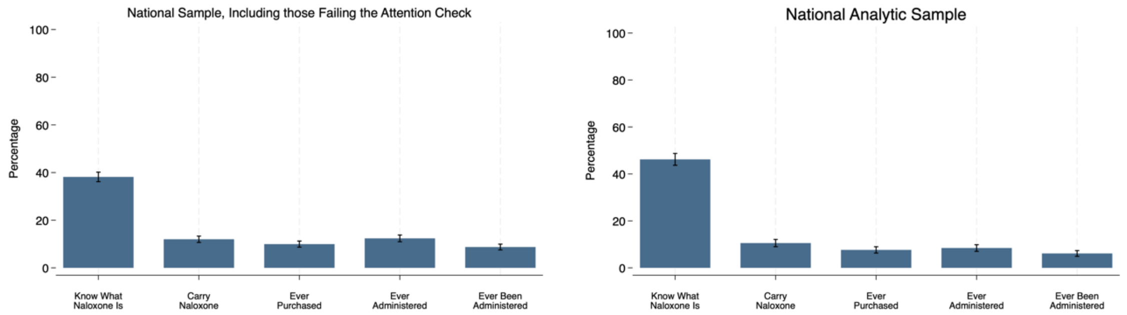


A. National Sample

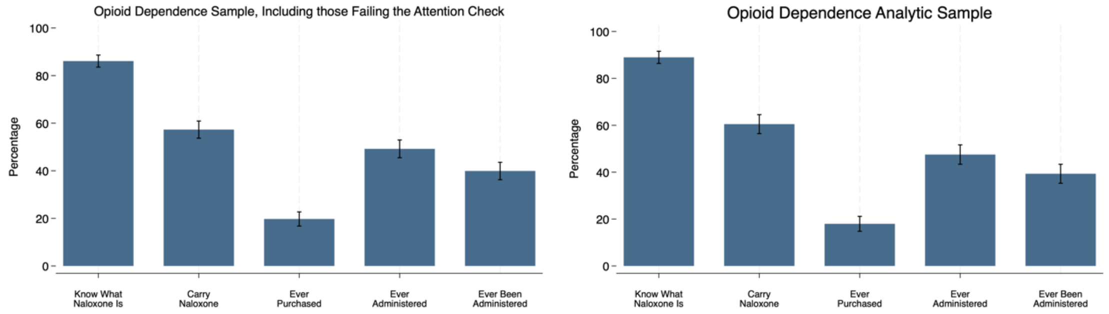


B. Respondents Reporting Opioid Dependence

*Notes: Panel A presents percentages and 95% confidence intervals for the full national sample (N=2290) and the analytic national sample (N=1,515); Panel B for full opioid dependence sample (N=726) and the analytic opioid dependence sample (N=562). People are coded as "carry naloxone" if they self-reported that they currently carry naloxone "occasionally" or "most or all of the time." The analytic sample results are identical to those included in the main paper and are included here for comparison purposes.*

© 2025 Jacobson M et al. *JAMA Network Open*.

eFigure 4. Naloxone Knowledge by Subgroups of Opioid Exposure

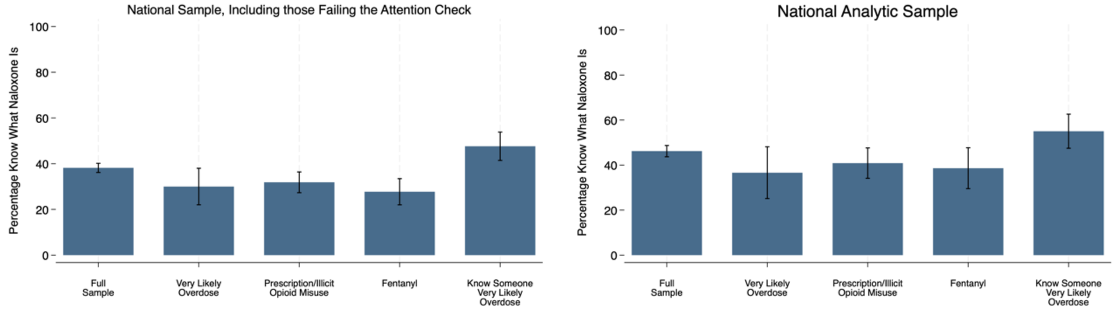


A. National Sample

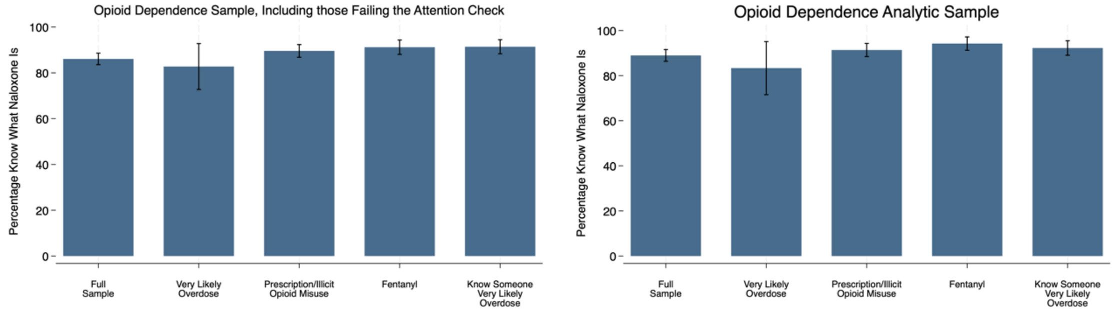


B. Respondents Reporting Opioid Dependence

*Notes: Panel A presents percentages and 95% confidence intervals for the "National Sample, including those Failing the Attention Check" and the national analytic sample. For the "National Sample, including those Failing the Attention Check," the columns report naloxone knowledge for (1) everyone (N=2,290), (2) those reporting that it is very likely that they will overdose from opioid use (N=130), (3) those reporting misuse of prescription opioids or use of illicit opioids with the past 12 months (N=408), (4) those reporting illicitly-made fentanyl use within the past 12 months (N=238) and (5) those reporting that they know someone who is very likely to overdose from opioid use (N=252).*

*Panel B presents the same statistics for the "Opioid dependence sample including those Failing the Attention Check," and the corresponding analytic sample. The sample sizes are 726 for the first column, 58 for the second column, 470 for the third column, 318 for the fourth column, and 325 for the final column.*

*For analytic sample numbers (national or opioid dependence), see the notes to Figure 2. The analytic sample results are identical to those included in the main paper and are included here for comparison purposes.*

© 2025 Jacobson M et al. *JAMA Network Open*.

eFigure 5. Proportion Currently Carrying Naloxone by Subgroup

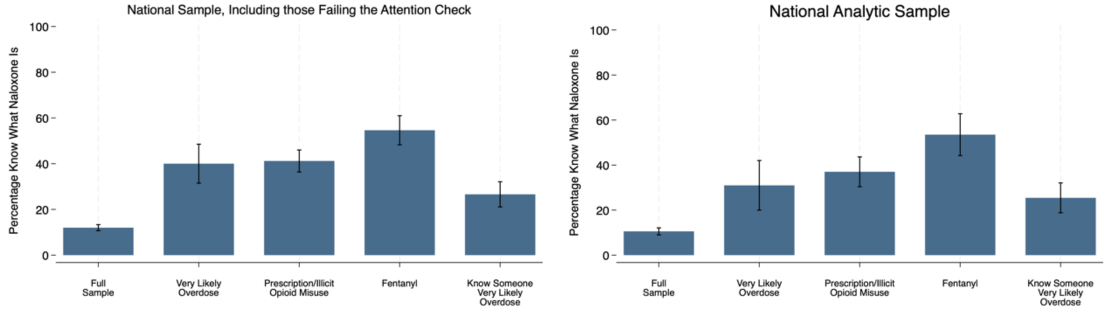


A. National Sample

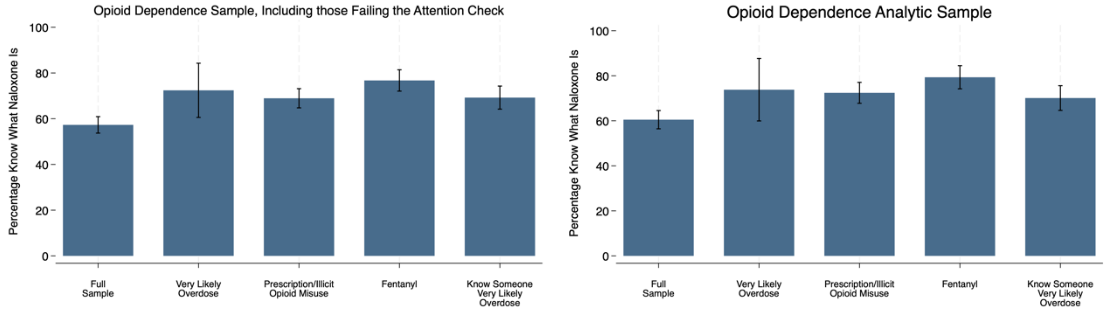


B. Respondents Reporting Opioid Dependence

*Notes: Currently carrying naloxone is defined as the respondent reporting that they currently carry naloxone "occasionally" or "most or all of the time."*

*Panel A presents percentages and 95% confidence intervals for the "National Sample, including those Failing the Attention Check" and the national analytic sample. For the "National Sample, including those Failing the Attention Check," the columns report naloxone knowledge for (1) everyone (N=2,290), (2) those reporting that it is very likely that they will overdose from opioid use (N=130), (3) those reporting misuse of prescription opioids or use of illicit opioids with the past 12 months (N=408), (4) those reporting illicitly-made fentanyl use within the past 12 months (N=238) and (5) those reporting that they know someone who is very likely to overdose from opioid use (N=252).*

*Panel B presents the same statistics for the "Opioid dependence sample including those Failing the Attention Check," and the corresponding analytic sample. The sample sizes are 726 for the first column, 58 for the second column, 470 for the third column, 318 for the fourth column, and 325 for the final column.*

*For analytic sample numbers (national or opioid dependence), see the notes to Figure 2. The analytic sample results are identical to those included in the main paper and are included here for comparison purposes.*

© 2025 Jacobson M et al. *JAMA Network Open*.

eAppendix 4. Internal Consistency of Responses

We checked whether responses were internally consistent to safeguard against inattention and false responses. We used only the nationally-representative sample for these tests. For our first test, we evaluated how people responded that they were personally impacted by opioids among respondents who self-reported no prescription opioid use in the past 12 months, no prescription opioid misuse in the past 12 months, and no use of non-prescription opioids in the past 12 months (N=1,107). eFigure 5 reports this sample's responses regarding how they have been personally impacted by opioids. Respondents could select more than one option for this question. 95.48% (95% CI: 94.26% - 96.71%) reported no impact while a small share (3.25% [95% CI: 2.21% - 4.30%]) reported that they struggled with dependence and 1.72% (95% CI: 0.95% - 2.48%) reported that they sought treatment. These latter answers would be consistent with opioid dependence issues over 12 months before the survey. The low rates of respondents in this sample claiming any prior impacts of opioids in their life is consistent with the absence of opioid use and misuse within the past 12 months.

Next, we evaluated the self-reported rates of overdose likelihood among respondents who self-reported no prescription opioid use in the past 12 months, no prescription opioid misuse in the past 12 months, no use of non-prescription opioids in the past 12 months, and reported that they have not been personally impacted by opioids (N=1,057). These selections do not imply that the respondent has not had past exposure to opioids, nor does it imply that they might not anticipate an overdose involving opioids if, for example, they frequently use illicit substances and are aware that illicitly-made fentanyl could potentially contaminate those substances. eFigure 6 shows that 92.90% (95% CI: 91.36% - 94.45%) of these respondents reported it was unlikely that they would overdose from opioid use. This high rate suggests that the responses were internally consistent.

© 2025 Jacobson M et al. *JAMA Network Open*.

eFigure 6. Personal Impacts of Opioid Use Among Respondents with No Prescription or Illicit Opioid Use within Past 12 Months (N=1,107)

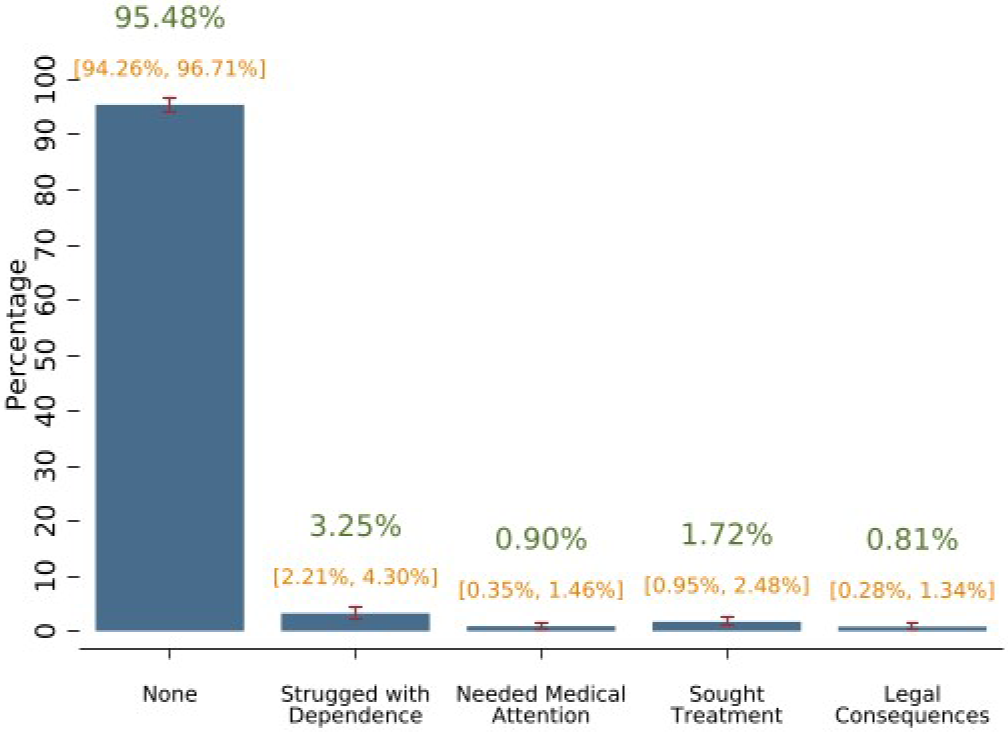


*Notes: Respondents could select "None" or at least 1 of the other options.*

© 2025 Jacobson M et al. *JAMA Network Open*.

eFigure 7. Self-Reported Likelihood of Overdosing from Opioid Use for Respondents with No Prescription or Illicit Opioid Use within Past 12 Months and No Personal Impact from Opioid Use (N=1,057)

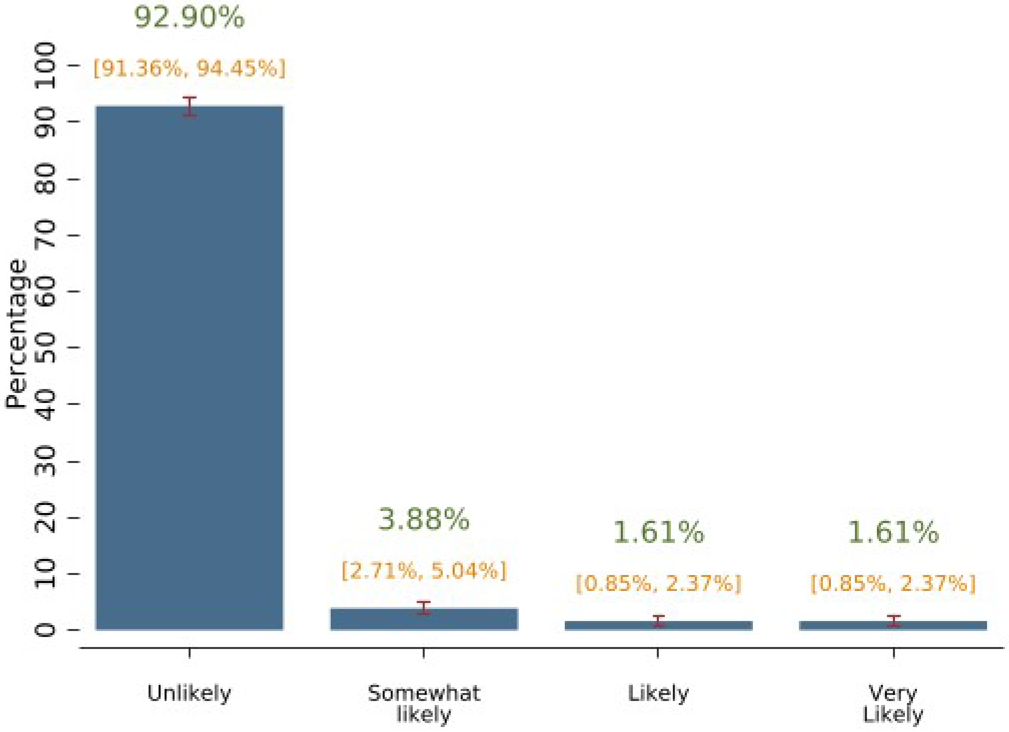


© 2025 Jacobson M et al. *JAMA Network Open*.

## **eAppendix 5.** Full Survey

**CT001** (consent)
This survey asks about your knowledge of and personal experiences with substance use in the United States. The survey is for a research study conducted by RAND and the University of Southern California. It includes sensitive questions about prior substance use. Your responses are confidential and the researchers receiving the data will not have access to any identifying information. Please do not include any personally identifying information in any of your responses. You can stop the survey at any time.
We know that this information is personal, but your answers are important to our understanding of substance use in the US. This survey should take 10 minutes of your time.

If the topics in
this survey bring up issues that you would like to discuss with someone. Please call the Substance Abuse and Mental Health Services (SAMHSA) helpline: 1-800-662-3457.

This study was
approved by the RAND Human Subjects Protection Committee and has also been reviewed by the USC Institutional
Review Board (IRB). The IRB is a research review board that reviews and monitors research studies to protect the rights and welfare of research participants. Contact the IRB if you have questions about your rights as a research participant or have complaints about the research. You can contact RAND's Human Subjects Protection Committee toll-free at (866) 697-5620 or by emailing hspcinfo@rand.org. When you contact the RAND Committee, please reference Study #2023-N0309. You can also contact USC's IRB at (323) 442-0114 or by email at hrpp@usc.edu. When you contact USC's IRB, please reference Study UP -24-00279.
1 I've read the information above and agree to proceed with the survey

IF CT001 = 1 THEN
|
Else
|
| **RS004_screenout** (screen out)
| Thanks for taking part.
|
| Unfortunately, you don't fit the required profile for this
| piece of market research.
|
| Exit the survey
|
End of if

Group of questions presented on the same screen
|
| DM001
| What is your gender?
| 1 Male
| 2 Female
| 3 Prefer to self-identify:
|



© 2025 Jacobson M et al. *JAMA Network Open*.

| **DM001_self** (gender self identify)
| What is your gender?
| STRING
|
End of group of questions

IF !checkGenderQuota(Sampletype, DM001) THEN
|
| **RS003_quota** (quota full)
| Thanks for taking part.
|
| Sorry, but we've received all the answers we need from respondents
| fitting your profile.
|
| Exit the survey
|
End of if

**DM002** (age)
What is your age?
RANGE 0..120

IF DM002 < 18 THEN
|
| **RS004_screenout** (screen out)
| Thanks for taking part.
|
| Unfortunately, you don't fit the required profile for this
| piece of market research.
|
| Exit the survey
|
End of if

IF !checkAgeQuota(Sampletype, DM002) THEN
|
| **RS003_quota** (quota full)
| Thanks for taking part.
|
| Sorry, but we've received all the answers we need from respondents
| fitting your profile.
|
| Exit the survey
|
End of if

**DM003** (education)



© 2025 Jacobson M et al. *JAMA Network Open*.

Which of the following best describes your education?
1 Did not attend high school
2 Left high school before graduation
3 Received a GED
4 High school graduate
5 Some college - no degree
6 Technical or Trade School - certificate or credential
7 College graduate – associate's degree
8 College graduate – bachelor's degree
9 College graduate – master's degree (MBA or other)
10 College graduate – advanced degree (JD, MD, PhD or other)

Group of questions presented on the same screen
|
| **DM004** (race)
| Which of the following best describes your race?
| 1 American Indian or Alaska Native
| 2 Asian American or Pacific Islander
| 3 Black
| 4 White
| 5 Other:
|
| **DM004_other** (race other)
| Which of the following best describes your race?
| STRING
|
End of group of questions

**DM005** (hispanic)
Are you Hispanic or Latino origin or descent?
1 Yes
2 No

**DM006** (political affiliation)
What would you consider your political party affiliation?
1 Republican
2 Democrat
3 Other

**DM009** (suffering from health issues)
Please indicate if you are currently suffering from, or have ever suffered from any of the following health issues:
1 Diabetes
2 Alcohol Dependence
3 Opioid Dependence
4 Cancer



© 2025 Jacobson M et al. *JAMA Network Open*.

5 Depression
6 Asthma
7 Cardiovascular disease
8 Chronic kidney disease
9 Chronic lung disease
10 None of the above

IF Sampletype = 2 THEN
|
| IF 3 IN DM009 THEN
| |
| Else
| |
| | **RS004_screenout** (screen out)
| | Thanks for taking part.
| |
| | Unfortunately, you don't fit the required profile for
| | this piece of market research.
| |
| | Exit the survey
| |
| End of if
|
End of if

**DM007** (state live)
What state do you live in?
99 I do not live in one of the 50 states or DC
1 Alabama (AL)
2 Alaska (AK)
4 Arizona (AZ)
5 Arkansas (AR)
6 California (CA)
8 Colorado (CO)
9 Connecticut (CT)
10 Delaware (DE)
11 District of Columbia (DC)
12 Florida (FL)
13 Georgia (GA)
15 Hawaii (HI)
16 Idaho (ID)
17 Illinois (IL)
18 Indiana (IN)
19 Iowa (IA)
20 Kansas (KS)
21 Kentucky (KY)
22 Louisiana (LA)



© 2025 Jacobson M et al. *JAMA Network Open*.

23 Maine (ME)
24 Maryland (MD)
25 Massachusetts (MA)
26 Michigan (MI)
27 Minnesota (MN)
28 Mississippi (MS)
29 Missouri (MO)
30 Montana (MT)
31 Nebraska (NE)
32 Nevada (NV)
33 New Hampshire (NH)
34 New Jersey (NJ)
35 New Mexico (NM)
36 New York (NY)
37 North Carolina (NC)
38 North Dakota (ND)
39 Ohio (OH)
40 Oklahoma (OK)
41 Oregon (OR)
42 Pennsylvania (PA)
44 Rhode Island (RI)
45 South Carolina (SC)
46 South Dakota (SD)
47 Tennessee (TN)
48 Texas (TX)
49 Utah (UT)
50 Vermont (VT)
51 Virginia (VA)
53 Washington (WA)
54 West Virginia (WV)
55 Wisconsin (WI)
56 Wyoming (WY)

IF DM007 = 99 THEN
|
| **RS004_geo** (geo)
| We are sorry...
|
| This survey is not accessible from your current geographic location
| .
|
| Exit the survey
|
End of if

DM008 := getRegion(DM007)



© 2025 Jacobson M et al. *JAMA Network Open*.

IF !checkRegionQuota(Sampletype, DM008) THEN
|
| **RS003_quota** (quota full)
| Thanks for taking part.
|
| Sorry, but we've received all the answers we need from respondents
| fitting your profile.
|
| Exit the survey
|
End of if

**NN001** (heard of narcan)
Have you heard of the overdose reversal medication naloxone, also sometimes known by its brand-name Narcan?
1 Yes
2 No

IF NN001 = 1 THEN
|
| NN002
| Naloxone is used to reverse the effects of
| 1 Opioid overdose (heroin, oxycodone, methadone)
| 2 Amphetamine overdose (Adderall)
| 3 Cocaine overdose
| 4 Any overdose
| 5 I don't know
|
End of if

IF NN002 != 1 THEN
|
| **NN003** (naxolone info)
| Naloxone can be administered to someone overdosing due to opioid use and potentially reverse the overdose
| and save the person's life.
|
End of if

**NN004** (used any prescription opiods)
In the past 12 months, have you used any prescription opioids?

Some examples include
Vicodin, Lortab, Norco, Zohydro ER, Hydrocodone, OxyContin, Percocet, Percodan, Roxicodone, Oxycodone
, Ultram, Ultram ER, Ultracet, Tramadol, Codeine, Tylenol with codeine (not over-the-counter Tylenol
), Avinza, Kadian, MS Contin, Morphine, Duragesic, Fentora, Fentanyl, Suboxone, Buprenorphine, Buprenorphine



© 2025 Jacobson M et al. *JAMA Network Open*.

plus naloxone, Opana, Oxymorphone, Demerol, Dilaudid, Hydromorphone, Exalgo, Methadone.
1 Yes
2 No

**NN005** (used any opioids that doctor did not direct you to use)
In the past 12 months, have you used any prescription opioids in a way that a doctor did not direct you to use, including: Using it without a prescription of your own Using it in greater amounts, more often, or longer than you were told to take it Using it in any other way a doctor did not direct you to use it?
1 Yes
2 No

**NN006** (used any non prescription opiods)
In the past 12 months, have you used any non-prescription opioids? Some examples include heroin or illicitly-made fentanyl.
1 Yes, I intentionally used non-prescription opioids
2 Yes, I unintentionally may have used non-prescription opioids
3 No, not that I am aware of

IF NN006 = 1 OR NN006 = 2 THEN
|
| **NN007** (ever used fentanyl)
| In the past 12 months, have you ever used illicitly-made fentanyl?
| 1 Yes, I have intentionally used illicitly-made fentanyl
| 2 Yes, I unintentionally may have used illicitly-made fentanyl
| 3 No, not that I am aware of
|
| **NN008** (first used opioids were)
| When you first used opioids for medical or for non-medical purposes, were they
| 1 Prescription opioids prescribed to you
| 2 Prescription opioids that were not prescribed to you
| 3 Non-prescription opioids (such as heroin or illicitly-made fentanyl)
|
End of if

**NN009** (someone you know been impacted)
Has someone you know been impacted by opioids in any of the following ways? I know someone that has…

Choose all that may apply
1 Struggled with an opioid addiction.
2 Needed medical attention because of an opioid addiction.
3 Sought treatment for an opioid addiction.
4 Faced police contact, arrest, or incarceration due to an opioid addiction.
5 Died of an overdose involving opioids
6 None of the above

**NN010** (personally been impacted by opioids)



© 2025 Jacobson M et al. *JAMA Network Open*.

Have you personally been impacted by opioids in any of the following ways? I have...

Choose all that may apply
1 Struggled with an opioid addiction.
2 Needed medical attention because of an opioid addiction.
3 Sought treatment for an opioid addiction.
4 Faced police contact, arrest, or incarceration due to an opioid addiction.
5 None of the above

**NN011** (how likely someone you know might overdose)
How likely do you think it is that someone you know might overdose from opioid use?
1 Unlikely
2 Somewhat Likely
3 Likely
4 Very Likely

**NN012** (how likely you will overdose)
How likely do you think it is that you might overdose from opioid use?
1 Unlikely
2 Somewhat Likely
3 Likely
4 Very Likely

**NN013** (ever administered naloxone)
We previously asked about your knowledge of naloxone, which is sometimes referred to as Narcan.

Have you ever administered naloxone to another person experiencing an overdose?
1 Yes
2 No

IF NN013 = 1 THEN
|
| **NN014** (when last administer naloxone)
| When did you last administer naloxone to another person experiencing an overdose?
| 1 Within the last month
| 2 Over a month ago but within the last year
| 3 Between 1 and 5 years ago
| 4 Over 5 years ago
|
| End of if
|
**NN015** (anyone ever administered naloxone to you)
Has anyone ever administered naloxone to you?
1 Yes
2 No



© 2025 Jacobson M et al. *JAMA Network Open*.

IF NN015 = 1 THEN
|
| **NN016** (when last administer naloxone to you)
| When did someone last administer naloxone to you?
| 1 Within the last month
| 2 Over a month ago but within the last year
| 3 Between 1 and 5 years ago
| 4 Over 5 years ago
|
End of if

**NN017** (previously carried or do you currently carry naloxone)
Have you previously carried or do you currently carry naloxone?
1 Yes, I have carried it in the past but do not now
2 Yes, I currently carry it occasionally
3 Yes, I currently carry it most or all of the time
4 No, I have never carried naloxone

**NN018** (aware narcan is available over the counter)
Are you aware that Narcan is now available over-the-counter, meaning it can be purchased directly, without asking a pharmacist to provide it?
1 Yes, I have seen it available over-the-counter in a store
2 Yes, but I have not seen it available over-the-counter in a store
3 No, I was not aware

**NN028** (ever purchased naloxone)
Have you ever purchased naloxone?
1 Yes
2 No

IF NN028 = 1 THEN
|
| Group of questions presented on the same screen
| |
| | **NN021** (how much did you pay for the naloxone over the counter)
| | How much did you pay the last time you purchased naloxone?
| |
| | Please round to the
| | nearest dollar
| | RANGE 1..1000
| |
| | **NN027** (quantity)
| | What quantity did you buy?
| | 1 1-Pack
| | 2 2-Pack
| | 3 Other
| |



© 2025 Jacobson M et al. *JAMA Network Open*.

| | **NN027_other** (quantity other)
| | What quantity did you buy?
| | STRING
| |
| End of group of questions
|
| End of if

IF NN017 = 1 OR NN017 = 2 OR NN017 = 3 THEN
|
| Group of questions presented on the same screen
| |
| | **NN019** (where obtain naloxone)
| | Where did you obtain the naloxone that you currently carry or previously carried?
| |
| |
| | Check all that apply
| | 1 From a pharmacist (not over-the-counter)
| | 2 From a clinic, hospital, or other medical setting
| | 3 At a harm reduction organization or public health department
| | 4 In the mail
| | 5 From a vending machine
| | 6 Provided by a friend, family member, or someone else.
| | 7 Over-the-counter at a retail outlet
| | 8 Other:
| |
| | **NN019_other** (where obtain naloxone other)
| | Where did you obtain the naloxone that you currently carry or previously carried?
| |
| |
| | Check all that apply
| | STRING
| |
| End of group of questions
|
| **NN020** (did you purchase the naloxone)
| Did you purchase the naloxone that you currently or previously carried?
| 1 Yes, purchased it.
| 2 No, received it free.
| 3 Have both purchased naloxone and received it free.
|
End of if

Group of questions presented on the same screen
|
| **NN023** (interest in experience write none)
| We are interested in your experience with naloxone. We would also like to make sure that respondents



© 2025 Jacobson M et al. *JAMA Network Open*.

| are reading and answering these questions carefully. Please ignore the choices and write "none" into
| the box next to "Other" below.
| 1 Strongly agree
| 2 Agree
| 3 Disagree
| 4 Strongly disagree
| 5 Other:
|
| **NN023_other** (interest in experience write none other)
| We are interested in your experience with naloxone. We would also like to make sure that respondents
| are reading and answering these questions carefully. Please ignore the choices and write “none” into
| the box next to “Other” below.
| STRING
|
End of group of questions

IF NN023_other = 'None' OR NN023_other = 'none' OR NN023_other = 'Non' OR NN023_other = 'non'
THEN
|
Else
|
| **RS004_quality** (quality)
| Thanks for taking part. Unfortunately, your answers were excluded from the survey. Due to this, Maximiles
| points cannot be awarded to your account for this survey.
|
| The possible reasons for
| this are: We already have your answers for this survey. A member of your household has already
| answered the survey using the same computer. Your answers do not pass quality verification checks.
| You are not in the required geographical location.
|
End of if

IF NN017 = 1 OR NN017 = 2 OR NN017 = 3 THEN
|
| Group of questions presented on the same screen
| |
| | **NN024** (why carry naloxone)
| | Why do/did you carry naloxone?
| |
| | Select all that apply
| | 1 So someone can administer it to me if I have an overdose
| | 2 So I or someone else can administer it to a friend or loved one in case they have an overdose
| | 3 So I or someone else can administer it to someone other than a friend or loved one in case they have an overdose
| | 4 Other:
| |
| | **NN024_other** (why carry naloxone other)



© 2025 Jacobson M et al. *JAMA Network Open*.

| | Why do/did you carry naloxone?
| |
| | Select all that apply
| | STRING
| |
| | End of group of questions
|
End of if

**NN025** (availability affected you)
Has the availability of naloxone affected you in any of the following ways?

Select all
that apply
1 I am more likely to use opioids for non-medical purposes because other people are carrying naloxone
2 I am more likely to use opioids for non-medical purposes because I am carrying naloxone
3 It has not affected me

IF NN017 = 4 THEN
|
| Group of questions presented on the same screen
| |
| | **NN026** (why not carry naloxone)
| | Why do you not carry naloxone?
| |
| | Select all that apply
| | 1 I did not know what it was prior to this survey
| | 2 It is unlikely that I will need to use naloxone
| | 3 Naloxone costs too much
| | 4 I worry about what people who see me purchasing or carrying naloxone will think
| | 5 I have tried to find naloxone, but I was unable to find it
| | 6 I am not sure how to obtain naloxone
| | 7 Other:
| |
| | **NN026_other** (why not carry naloxone other)
| | Why do you not carry naloxone?
| |
| | Select all that apply
| | STRING
| |
| | End of group of questions
|
End of if

HL_intro
We would now like to ask some hypotheticals about your willingness to purchase naloxone.



© 2025 Jacobson M et al. *JAMA Network Open*.

if HL008_009_randomizer = 1 then
|
| **HL008** (amount would pay for product)
| It is possible to purchase Narcan at a pharmacy without a prescription. What is the maximum amount that
| you would be willing to pay to purchase a two-dose kit of Narcan from a pharmacist?
|
|
| Move the slider to the maximum amount that you would pay for the product. If, for example, you move
| the slider to $50 that means you would purchase the product if it cost $50 or less. If the product cost
| more than $50 though, you would not purchase it. All costs represent your total out-of-pocket payment
| after taxes and accounting for any insurance coverage.
|
| If you are not willing to
| pay anything, then move the slider to $0. The maximum amount on the slider is $250.
|
Else
|
| **HL009** (amount would pay for product)
| It is possible to purchase Narcan as an over-the-counter drug. What is the maximum amount that you would
| pay to purchase an over-the-counter two-dose kit of Narcan?
|
| Move the slider to the
| maximum amount that you would pay for the product. If, for example, you move the slider to $50 that
| means you would purchase the product if it cost $50 or less. If the product cost more than $50 though
| , you would not purchase it. All costs represent your total out-of-pocket payment after taxes and accounting
| for any insurance coverage.
|
| If you are not willing to pay anything, then move the
| slider to $0. The maximum amount on the slider is $250.
|
End of if

if HL010_randomizer = 1 then
|
| **HL010a** (public attention to the opioid crisis no group mentioned)
| Opioid-involved overdose deaths increased about 10-fold since 1999, from about 8,000 to 80,000 deaths
| annually. More than twice the number of people die from an opioid-involved overdose than a motor vehicle
| crash.
|
| In your opinion, public attention to the opioid crisis and spending on policies| to decrease overdose deaths,
| such as more opioid use treatment access and more naloxone access, is
| 1 Too much
| 2 Just right
| 3 Not enough
|



© 2025 Jacobson M et al. *JAMA Network Open*.

elseif HL010_randomizer = 2 then
|
| **HL010b** (public attention to the opioid crisis black american indian)
| Opioid-involved overdose deaths increased about 10-fold since 1999, from about 8,000 to 80,000 deaths
| annually. Black and American Indian/Alaska Native individuals currently have the highest overdose death
| rates.
|
| In your opinion, public attention to the opioid crisis and spending on policies to decrease overdose deaths,
| such as more opioid use treatment access and more naloxone access, is
| 1 Too much
| 2 Just right
| 3 Not enough
|
Else
|
| **HL010c** (public attention to the opioid crisis white)
| Opioid-involved overdose deaths increased about 10-fold since 1999, from about 8,000 to 80,000 deaths
| annually. White and American Indian/Alaska Native individuals have had the highest overdose death rates
| during the opioid crisis.
|
| In your opinion, public attention to the opioid crisis and spending on policies to decrease overdose deaths,
| such as more opioid use treatment access and more naloxone access, is
| 1 Too much
| 2 Just right
| 3 Not enough
|
End of if

IF HL010a = 1 OR HL010a = 2 OR HL010b = 1 OR HL010b = 2 OR HL010c = 1 OR HL010c = 2 THEN
|
| Group of questions presented on the same screen
| |
| | **HL012** (why think the government should spend more)
| | Why don't you think the government should spend more on and/or give more attention to the opioid crisis
| | ?
| |
| | Choose all that apply
| | 1 The country has other problems that deserve more attention
| | 2 Opioid use is a choice and people should just not use opioids
| | 3 Spending more money won't address the problem
| | 4 Other:
| |
| | **HL012_other** (why think the government should spend more other)
| | Why don't you think the government should spend more on and/or give more attention to the opioid crisis
| | ?
| |
| | Choose all that apply



© 2025 Jacobson M et al. *JAMA Network Open*.

| | STRING
| |
| End of group of questions
|
End of if

**HL013** (maximum dollars you would be willing to contribute)
In 2022, 80,000 people died of an overdose involving opioids or about 22 out of every 100,000 people . Imagine that we could help people pay for naloxone and we knew with certainty that this program would reduce the rate of opioid overdose deaths to 21 out of every 100,000 people, saving 1 out of every 100,000 people in the United States. Such a program would be costly. What is the maximum (in dollars ) that you personally would be willing to contribute this year to run this program?

If
, for example, you move the slider to $50 that means you would be willing to contribute an amount up to and including $50. If the policy cost you $50 or less, you would be in favor of it. If the policy cost you over $50, then you would oppose it.

You can select $0, and the maximum amount on the slider is $250.

**HL014** (opioid addiction is a choice)
Do you agree/disagree with the following statement: Opioid addiction is a choice.
1 Strongly agree
2 Agree
3 Neither agree nor disagree
4 Disagree
5 Strongly disagree

**HL015** (most people found misusing opioids should)
Do you think most people found misusing opioids not prescribed by a doctor should:
1 Be ordered to undergo treatment without jail time
2 Serve jail time
3 Neither mandated treatment or jail time
4 Don't know

**HL016** (is there an effective treatment)
To the best of your knowledge, is there an effective treatment for opioid addiction?
1 Yes
2 No
3 Don't know

**HL017** (should prisons provide moud)
Medication for opioid use disorder (MOUD) – including buprenorphine, methadone, and naltrexone -- is often considered an important part of treatment for opioid use disorders.



© 2025 Jacobson M et al. *JAMA Network Open*.

Many people
in prisons and jails have opioid use disorders. Should prisons provide MOUD to prisoners?
1 Yes
2 No
3 Don't know

**HL018** (support a policy that gives naloxone to prisoners)
Would you support a policy that gives naloxone to prisoners with a history of opioid use disorder upon their release?
1 Yes
2 No
3 Don't know

**CS_003** (comments)
Do you have any other comments on the survey? Please type these in the box below. (If you have no comments
, please click next to complete this survey.)
STRING

endtime := date("Y-m-d H:i:s")

IF Sampletype = 1 THEN
|
| **RS001_national** (completed national representative)
| Thanks for completing the survey!
|
Else
|
| **RS002_opioid** (completed opioid)
| Thanks for completing the survey!
|
End of if



© 2025 Jacobson M et al. *JAMA Network Open*.
