# Supplementary material for: Naloxone Knowledge, Carrying, Purchase, and Use
Source: JAMA Netw Open. 2025 Mar 3;8(3):e2462698. doi: 10.1001/jamanetworkopen.2024.62698 (PMC11877168; doi:10.1001/jamanetworkopen.2024.62698)
Supplement: Supplement 2. — Data Sharing Statement [file jamanetwopen-e2462698-s002.pdf]

Data Sharing Statement

Jacobson. Naloxone Knowledge, Carrying, Purchase, and Use. *JAMA Netw Open*. Published March 03, 2025. doi:10.1001/jamanetworkopen.2024.62698

Data
Data available: Yes
Data types: Deidentified participant data
How to access data: https://dataverse.harvard.edu/
When available: beginning date: 05-01-2025

Supporting Documents
Document types: Statistical/analytic code, Other (please specify)
Additional Information: codebook
How to access documents: https://dataverse.harvard.edu/
When available: beginning date: 05-01-2025

Additional Information
Who can access the data: Anyone requesting data
Types of analyses: any purpose
Mechanisms of data availability: download from site
